# Absorption Spectra of AlGaN/GaN Terahertz Plasmonic Crystals—Experimental Validation of Analytical Approach

**DOI:** 10.3390/nano14181502

**Published:** 2024-09-16

**Authors:** Maksym Dub, Pavlo Sai, Pawel Prystawko, Wojciech Knap, Sergey Rumyantsev

**Affiliations:** 1 Institute of High Pressure Physics, Polish Academy of Sciences, 01-142 Warsaw, Poland; sajpasha@gmail.com (P.S.); pprysta@unipress.waw.pl (P.P.); knap.wojciech@gmail.com (W.K.); roumis4@gmail.com (S.R.); 2 Center for Terahertz Research and Applications (CENTERA), Centre for Advanced Materials and Technologies (CEZAMAT), Warsaw University of Technology, 02-822 Warsaw, Poland

**Keywords:** plasmonic crystal, AlGaN/GaN, THz Fourier spectroscopy, grating gate

## Abstract

Absorption spectra of AlGaN/GaN grating-gate plasmonic crystals with a period from 1 µm to 2.5 µm were studied experimentally at *T* = 70 K using Fourier-transform infrared spectrometry. The plasmonic crystals exhibit distinct absorption lines of various plasmon harmonics across the 0.5 to 6 THz frequency range, tunable by gate voltage. Cumbersome and time-consuming electromagnetic simulations are usually needed to interpret or predict the grating-gate crystal spectra. In this work, we examine an analytical model and show that it can successfully describe the majority of existing experimental results. In this way, we demonstrate a new analytical platform for designing plasmonic crystals for THz filters, detectors, and amplifiers.

## 1. Introduction

Two-dimensional (2D) plasmons (collective oscillations of electron density) in semiconductor periodic structures have attracted the attention of the scientific community since the 1980s [1,2]. It was demonstrated theoretically and experimentally that they can be used as tunable detectors [3,4,5,6,7], broadband emitters [8,9], and potentially as tunable terahertz filters (absorbers) [10,11]. One of the simple ways to fabricate the 2D periodic structure is based on the heterostructure field effect transistor with periodic multiple gates. For AlGaAs/GaAs and AlGaN/GaN systems, the characteristic frequencies of the plasmons in this kind of grating-gate structure, with the period of micron size, lie in the terahertz frequency range, with the frequency tunable by the gate voltage. This provides one of the solutions for achieving the challenging terahertz frequency band using simple optical photolithography. Two-dimensional plasmons in a single cavity and periodic structures have been studied in multiple publications. However, it was also realized that periodic structures behave differently from the single plasmonic cavity devices and should be considered as lateral plasmonic crystals [11].

In a recent article [12], the AlGaN/GaN plasmonic crystals based on 2D electron gas and grating-gate structures were analyzed experimentally and by rigorous electrodynamic simulation. It was shown that the experimental absorption spectra with pronounced resonant features were very well described by these simulations. It was also demonstrated that the known analytical single cavity approach (see, for example, review [13]) did not provide correct resonance frequencies for the grating-gate structures of plasmonic crystals. While attempting to analyze the plasmonic crystals analytically, the most common approach is to calculate the angular plasma frequency as

$$w_{n}=q_{n}s_{g},$$

where *q_n_* is the wave vector, and *s_g_* is the plasma wave velocity taken to be equal to the velocity of the gated plasmon, which in the case of a thin barrier layer is given by [13]:

(1) $$s_{g}=\sqrt{\frac{4\pi e^{2}n_{g}d}{m^{*}\varepsilon }}.$$

Here, *e* is the elemental charge, *n_g_* is the 2D concentration under the gate, *d* is the barrier thickness, m* is the effective electron mass, and *ε* is the barrier material dielectric constant. The wave vector is usually taken equal to *q_n_* = 2*πn*/*P*, where *n* = 1, 2, 3, …, and *P* is the period [14,15,16,17,18,19].

However, this approach overlooks the properties of the ungated regions, which can make substantial contributions to the plasmon spectrum. As was shown in ref. [12], the rigorous electrodynamic simulations reproduce the plasmonic crystals behavior correctly. However, these simulations are complicated and time-consuming.

A simple analytical model is desired to analyze and design the plasmonic crystals with the required properties. This kind of model was proposed in ref. [20] and further developed in ref. [21]. However, its validity was never verified experimentally.

In this work, we present experimental studies of the THz properties of AlGaN/GaN grating-gate structures (with the period varying from 1 µm to 2.5 µm), which showed gate voltage tunable plasmonic resonances in the 0.5 THz to 6 THz frequency range.

We used the model in [21] to analyze the properties of AlGaN/GaN terahertz plasmonic crystals of different geometries. This includes the analysis of the structures fabricated in our laboratory as well as the data from the published works. We show that the model describes the experimental results well and can be used to design the plasmonic crystals with the required properties for THz filters, detectors, and amplifiers.

## 2. Device Fabrication

To fabricate the AlGaN/GaN grating-gate plasmonic crystals, we used two types of AlGaN/GaN heterostructures: commercially available (SweGaN, Linköping, Sweden) [22]) and grown in the Institute of High Pressure Physics (IHPP). The SweGaN structures were grown by the metalorganic vapor phase epitaxy (MOVPE) method on a 4-inch diameter, 500 µm-thick SiC substrate. The semiconductor stack consists of a 2.4 nm GaN cap, a 20.5 nm Al_0.25_Ga_0.75_N barrier, and a 255 nm GaN buffer grown directly on a 62 nm-think AlN nuclear layer on SiC substrate. The heterostructures in the Institute of High Pressure Physics PAS were also grown by MOVPE within a closed coupled showerhead 3 × 2 inch Aixtron reactor (Aixtron, Herzogenrath, Germany). This epi-structure consists of a 2 nm GaN cap, a 20 nm Al_0.28_Ga_0.72_N barrier, a 1 nm AlN layer, and a 3 µm GaN buffer. This structure was grown directly on a 40 nm-thick AlN nucleation layer situated a top of a 500 µm 6H-SiC substrate.

To isolate a device on the wafer, shallow 150 nm mesas were etched using Inductively Coupled Plasma-Reactive Ion Etching (ICP-RIE) equipment (Oxford Instruments, Bristol, UK). A thermal evaporation process was employed to fabricate ohmic contacts, using a metal stack consisting of Ti/Al/Ni/Au with respective layer thicknesses of 150/1000/400/500 Å. Subsequently, rapid thermal annealing (RTA) was performed at 800 °C for 60 s in a nitrogen atmosphere to minimize contact resistance to 1 Ω·mm.

**Figure 1:**
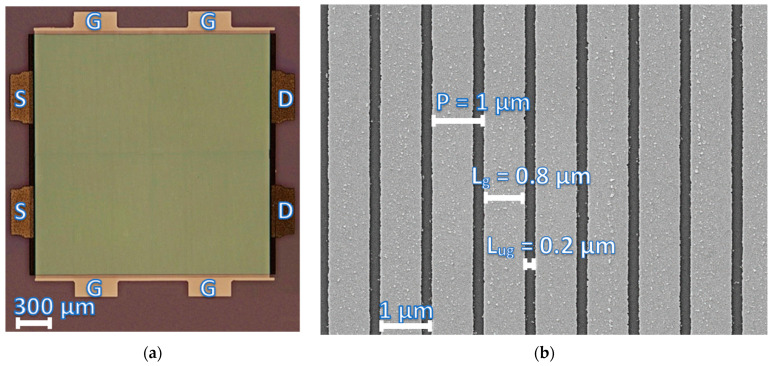
Optical microscope (**a**), and scanning electron microscope (**b**) images of one of the studied devices. Where S—source, G—gate and D—drain.

Schottky contacts were formed by the thermal evaporation of Ni/Au (150/350 Å) and metal lift-off. Figure 1 shows the optical (a) and scanning electron (b) microscope images of one of the fabricated devices with gated and ungated regions of *L_g_* = 0.8 µm and *L_ug_* = 0.2 µm, respectively. The total area of the structures was 2 × 2 mm^2^, with the total number of gates up to ~1695 fingers. For different devices, the period *P*, of the grating gates varied from 1 µm to 2.5 µm with a gate width from 0.5 µm to 1.6 µm. Fabrication of such a large area field effect transistor with a reasonably small gate-leakage current is a very challenging task. We used a special procedure for surface preparation before the electron beam lithography and metal deposition to minimize the gate-leakage current (see ref. [12] for more details).

## 3. Experiment Details

The transmission spectra of grating-gate structures were measured using a Fourier-transform infrared (FTIR) vacuum spectrometer (Vertex 80v, Bruker, Billerica, MA, USA), integrated with a continuous flow liquid nitrogen cryostat (Optistat CF-V by Oxford Instrument, Abingdon, UK). To minimize the optical losses, the original cryostat windows were substituted with polymethylpentene (TPX) windows. The spectrometer setup comprised a cryogenically cooled silicon bolometer (General Purpose 4.2 K Bolometer System, IRLabs, Tucson, AZ, USA) with a solid-state silicon beam splitter and an external water-cooled high-power Hg-arc lamp as a source (Bruker, Optik GmbH, Billerica, MA, USA). During the measurements, in fast scanning mode, the mirror moved with the frequency of 5 kHz, averaging interferograms over 100 scans within a double-scan interferogram regime. To minimize the Fabry–Pérot interferences stamping from the optically thick 500 µm SiC substrate, a spectrum resolution of 4 cm^–1^ (0.12 THz) was utilized. To reduce oversaturation of the Si-bolometer a 1.5 mm aperture was placed directly after the sample, permitting electromagnetic radiation transmission solely through the grating-gate active region. All measurements were conducted at *T* = 70 K. The input and output current-voltage characteristics of the grating-gate structures were measured inside the FTIR spectrometer using a Keysight B2902A Precision Source/Measure Unit (Keysight, CA, USA).

## 4. Results and Discussion

**Figure 2:**
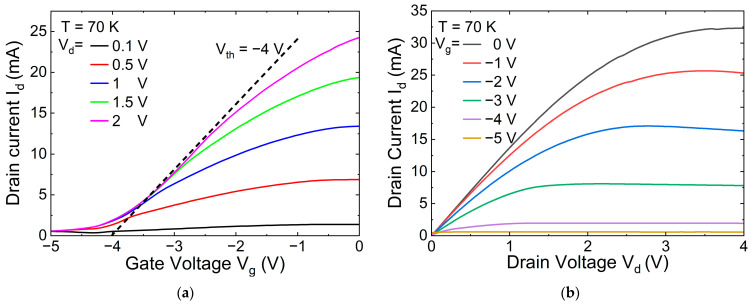
Input (**a**) and output (**b**) current-voltage characteristics of the representative AlGaN/GaN grating-gate plasmonic crystal structure at different drain and gate voltages *V_d_* and *V_g_*, respectively. Threshold voltage *V_th_* ≈ −4 V.

Figure 2 shows the transfer and output current-voltage characteristics of one of the transistors. As seen, despite the extremely high gate area of more than 2 × 2 mm^2^, the transistor behaves normally, i.e., the gate effectively controls the current with a reasonably small subthreshold current, which is determined by the gate leakage current.

**Figure 3:**
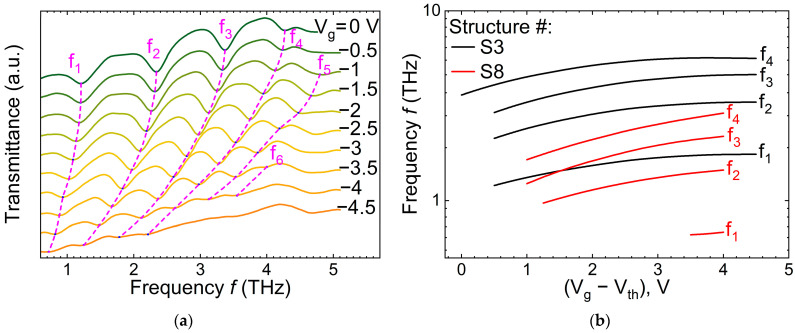
(**a**) Transmission spectra for the S2 structure at different gate voltages (shown with offset). The dashed lines are guides for eyes indicating the shift in the plasmon resonance frequencies *f_n_* = *ω_n_*/2*π*, where the typical absolute value of the transmittance at *f* = 1 THz is ~0.15. (**b**) Plasmon resonance frequencies as a function of the gate voltage overdrive *V_g_*–*V_th_* for the plasmonic crystals with different periods of *P* = 1 µm (structure S3, IHPP) and *P* = 2.5 µm (structure S8, SweGaN) and for different harmonics *n*.

Figure 3a shows examples of the transmission spectra at different gate voltages for a representative device. Up to five harmonics of plasmon resonances are clearly seen as absorption lines, which red shift with the negative gate voltage increase. Depending on the gate voltage and harmonic number, the resonance frequencies spread from ~0.7 to above 4 THz. Figure 3b shows the plasmon resonance frequencies as a function of the gate voltage overdrive *V*_g_–*V*_th_ for the plasmonic crystals with different periods of *P* = 1 µm and *P* = 2.5 µm. As seen, the resonance frequencies cover more than a decade from about 0.5 THz to 6 THz. This makes the design of the wide band terahertz spectrometer without moving mechanical parts promising. However, the analytical model is strongly desirable to predict the plasmonic crystal behavior as a function of the grating size, structure parameters, and gate voltage.

This kind of analytical model of the plasmonic crystal was proposed in ref. [20] and further developed in ref. [21]. It was shown that the resonance frequencies can be found as the solutions to the following equation:

(2) $$cos\frac{\omega_{n}L_{1}}{s_{1}}cos\frac{\omega_{n}L_{2}}{s_{2}}-\frac{s_{1}^{2}+s_{2}^{2}}{2s_{1}s_{2}}sin\frac{\omega_{n}L_{1}}{s_{1}}sin\frac{\omega_{n}L_{2}}{s_{2}}=1,$$

where *L*_1_ and *L*_2_ are the lengths of the plasmonic cavities, and *s*_1_ and *s*_2_ are the corresponding plasma wave velocities. Taking *L*_1_, *s*_1_ and *L*_2_, *s*_2_ as characteristics of the gated and ungated parts of the plasmonic crystal, we can estimate the plasma wave resonance frequencies *ω_n_*. In fact, for given structural parameters, Equation (2) yields two solutions of close frequencies, which are the so-called bright and dark modes [21]. Since these frequencies are close, we take only the lowest frequency for the analysis.

The ungated plasma wave velocities were calculated as [13]

(3) $$s_{ug}=\sqrt{\frac{4e^{2}n_{ug}L}{\left(\varepsilon +1\right)m^{*}}},$$

where *n_ug_* is the concentration in the ungated parts of the structure.

We measured the transmission spectra for six AlGaN/GaN plasmonic crystals and calculated their resonance frequencies *f_n_* = *ω_n_*/2*π* based on Equation (2). We also calculated the resonance frequencies for the known published experimental results.

Table 1 shows the parameters of the plasmonic crystals studied in the current work and in several publications along with the experimental and theoretical resonance plasma wave frequencies. All the experimental data from the published papers are for the temperatures T < 90 K.

**Figure 4:**
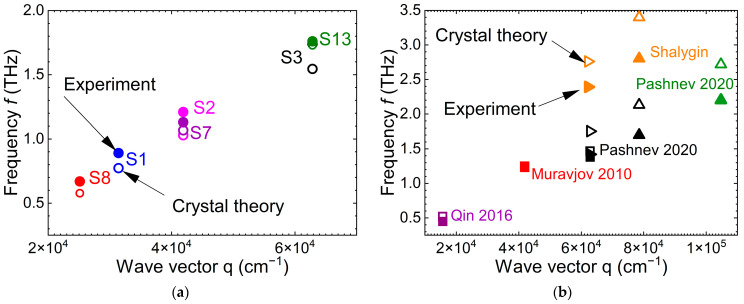
The experimental (solid symbols) frequencies of the first harmonic in AlGaN/GaN plasmonic crystals and those calculated using Equation (2) (open symbols) for the structures experimentally studied in the current work (**a**) and from the publications (**b**) [17,18,19,23,24]. The wave vector is taken to be equal to *q* = 2*π*/*P*.

Figure 4 summarizes these calculations for zero gate voltage for the structures in the current work (a) and for the experimental results from the other publications (b).

As seen from the table and Figure 4, the theory describes well the experimental results. However, one can mention that the discrepancy between the experiment and the model increases with the frequency increase. At high frequencies (high wave vectors), the model gives frequencies that are somewhat higher than the experimental ones. The main reasons for this discrepancy are probably that the model assumes two gated cavities (not gate and ungated as in the experiments) and zero losses, i.e., the absence of momentum relaxation. The model does not take into account the radiative losses as well. It is natural to assume that the radiative losses increase with the frequency increase.

It is also important to note that, for the same wave vector (period of the grating-gate structure), the resonance frequency may differ substantially. This is an indication that, contrary to some publications, not only the period but also the gated and ungated parts separately are important to consider to define the resonance frequency.

**Figure 5:**
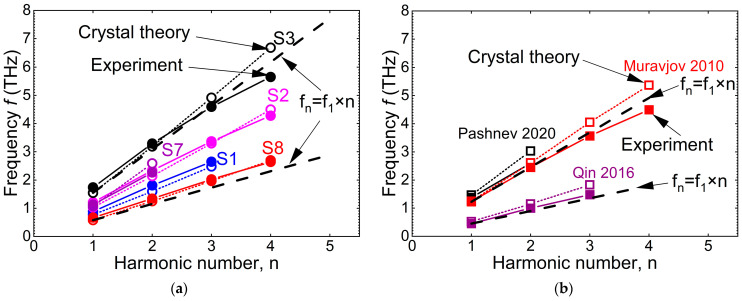
Experimental (solid symbols) frequencies as a function of the *f* the first harmonic number in AlGaN/GaN plasmonic crystals and those calculated using Equation (2) (open symbols) for the structures experimentally studied in the current work (**a**) and from the publications (**b**) [17,19,24]. The dashed lines in Figure 5 follow the dependence of *f_n_* = *f*_1_ × *n*, where *n* is the harmonic number.

Figure 5 summarizes the results for the frequency dependence on the harmonic number. As seen, the model describes the experiments well for higher harmonics as well. However, the discrepancy between the experiment and the theory increases with the harmonic number (frequency) increase.

The dashed lines in Figure 5 follow the dependence of *f_n_* = *f*_1_ × *n*, where *n* is the harmonic number. One can see that the experiment and the theory follow this rule rather well. The experimental values of the high harmonics were somewhat lower than expected.

Comparing Figure 4 and Figure 5, one can see that the higher the frequency the higher the discrepancy between the experiment and the model. As already mentioned above, one of the possible reasons for that can be the increasing losses with the frequency increase.

**Figure 6:**
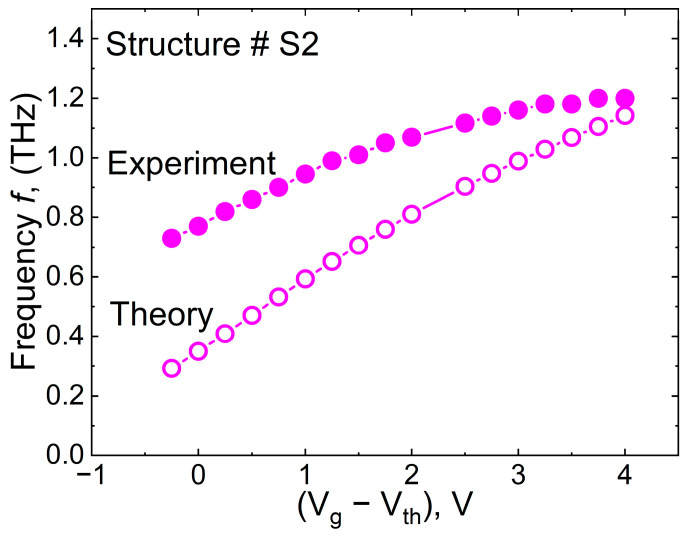
Frequency of the first harmonic as a function of the gate voltage overdrive (*V_g_*–*V_th_*) for the S2 structure. The solid symbols show the experimental data. The open symbols are calculated using Equation (2).

Figure 6 compares the experimental and theoretical gate voltage dependences of the first resonant harmonic for one of the structures (S2). The concentration under the gate was calculated as [25]

(4) $$n_{g}=\frac{c\eta kT}{e^{2}}ln\left[1+\frac{e\left(V_{g}-V_{th}\right)}{\eta kT}\right]$$

where *c*—gate capacitance per unit area, *η*—is the ideality factor, and *k*—Boltzmann constant.

As seen, the discrepancy between the experiment and the model increases while approaching the threshold voltage. One possible reason is that there is not good enough control over the concentration by the majority of the gates.

As seen in Figure 2 the current-voltage characteristics demonstrate a small subthreshold current indicating a small gate leakage current. However, in order to achieve the small gate leakage current, we do not need all the gates to be good. The studied structures contained more than 1500 gates. If just a few of them are good, the other imperfect gates do not provide leakage current, because the current flow to the drain and source is blocked by the other good gates. In other words, the small gate leakage current is not an indication of the high quality of all gates; they may not control the concentration perfectly, and Equation (4) yields underestimated concentration values at large negative gate voltages. The real concentration under the gate can be estimated using the discussed model [20,21].

The concentration at the threshold predicted by Equation (4) is *n*_g_ = 7.6 × 10^11^ cm^–2^. The concentration required to fit the experimental data at *V*_g_ = *V*_th_ is about 4 times higher *n*_g_ = 3.5 × 10^12^ cm^–2^, which should be considered as a real one for the majority of the gates. This large discrepancy between the estimate based on the current-voltage characteristics and the estimate based on the plasmon analysis cannot be explained by the inaccuracy of the estimates. Therefore, we believe that for the reasons discussed above, the concentration estimated from the plasmonic properties should be considered as a real one for the majority of the gates.

## 5. Conclusions

We experimentally studied the terahertz properties of AlGaN/GaN grating-gate crystals and demonstrated that structures with a period from 1 µm to 2.5 µm provide absorption lines tuned by the gate voltage within the frequency band from 0.5 to 6 THz. The analysis of our results as well as the results of other authors showed that the analytical model [20,21] describes the experimental data well and can be used to design terahertz filters, detectors, and amplifiers with the desired parameters.

## Figures and Tables

**Table 1:** Parameters of AlGaN/GaN grating-gate plasmonic crystals and resonance frequencies obtained from measured absorption spectra and calculated using Equation (2) (in parentheses).

Sample ID or Ref.	*P*, µm	*L_g_*, µm	*L_ug_*, µm	*d,* nm	*n_g_*, 10^12^ cm^−2^	*n_ug_*, 10^12^ cm^−2^	Frequency *f*_n_ (THz), Experiment (Theory)			
							Harmonic Number			
							1	2	3	4
Current Work										
S1 (IHPP)	2	1.6	0.4	22	7	10	0.89 (0.77)	1.81 (1.6)	2.65 (2.48)	
S2 (IHPP)	1.5	1.2	0.3				1.21 (1.03)	2.34 (2.14)	3.37 (3.3)	4.28 (4.5)
S3 (IHPP)	1	0.8	0.2				1.74 (1.55)	3.29 (3.19)	4.59 (4.92)	5.65 (6.68)
S7 (SweGaN)	1.5	0.9	0.6	23	5.5	8	1.13 (1.07)	2.27 (2.59)		
S8 (SweGaN)	2.5	1.8	0.7				0.67 (0.58)	1.34 (1.25)	2.02 (1.97)	2.64 (2.7)
S13 (SweGaN)	1	0.5	0.5				1.76 (1.73)			
Published results										
[17]	1	0.8	0.2	21.5	6.5	8.7	1.38 (1.47)	2.59 (3.03)		
[17]	0.8	0.44	0.36				1.70 (2.13)			
[17]	1	0.53	0.47				1.42 (1.75)			
[23]	0.6	0.35	0.25	21.5	6.5	8.7	2.20 (2.72)			
[18]	0.8	0.39	0.40	42	8	11.5	2.80 (3.40)			
[18]	1.01	0.49	0.51				2.39 (2.76)			
[19]	1.5	1.15	0.35	30	7.5	7.5	1.23 (1.25)	2.45 (2.61)	3.57 (4.05)	4.50 (5.37)
[24]	4	2.7	1.3	25	10	10	0.45 (0.52)	1.00 (1.15)	1.48 (1.83)	

## Data Availability

The data that support the findings of this study are available from the corresponding author upon reasonable request.
